# From Storytime to Success: Prospective Longitudinal Associations Between Toddler Literacy Enrichment and Long-Term Student Engagement in a Millennial Birth Cohort of Boys and Girls

**DOI:** 10.3390/jintelligence13060066

**Published:** 2025-06-10

**Authors:** Nairy Kazandjian, Kianoush Harandian, Stéfanie Routhier-Guilmette, Marie-Michèle Dufour, Isabelle Archambault, Linda S. Pagani

**Affiliations:** 1 School of Psychoeducation, University of Montreal, Montreal, QC H3T 1J4, Canada; nairy.kazandjian@umontreal.ca (N.K.); kianoush.harandian@umontreal.ca (K.H.); marie-michele.dufour@umontreal.ca (M.-M.D.); isabelle.archambault@umontreal.ca (I.A.); 2 School Environment Research Group, University of Montreal, Montreal, QC H3T 1J4, Canada; stefanie.routhier-guilmette@umontreal.ca; 3 Sainte-Justine’s Pediatric Hospital Research Center, University of Montreal, Montreal, QC H3T 1J4, Canada

**Keywords:** early literacy stimulation, literacy, academic engagement, child development, parental involvement, school readiness, crystallized intelligence

## Abstract

Cross-sectional research suggests associations between enrichment and cognitive skills in toddlerhood. There are no prospectively designed longitudinal studies that investigate the link between early home literacy activities and subsequent mechanisms that explain the putative cognitive benefits. This study tests long-term associations between toddler literacy enrichment and later student engagement across key academic transitions, from kindergarten to the end of high school. Using the Quebec Longitudinal Study of Child Development (QLSCD) population-based birth cohort data, we examined whether parent-reported experiences of shared reading, looking at picture books or illustrated stories, and pretend writing at age 2 years predict later teacher- and self-reported student engagement at ages 6, 12, and 17 years. The results from multiple regression models, stratified by sex and adjusted for pre-existing and concurrent child and family characteristics, revealed significant associations between early literacy enrichment and later engagement. For boys and girls, literacy enrichment in toddlerhood predicted increases in classroom engagement from kindergarten to the end of high school. These findings highlight the lasting influence of early literacy exposure on subsequent learning-related behaviors, both in and beyond the classroom. They underscore the importance of promoting enrichment in early childhood as a family strategy toward individual readiness to learn, a cornerstone of crystalized intelligence.

## 1. Introduction

Student engagement represents a key predictor of academic success, well-being, and future life outcomes (Symonds et al. 2023). It encompasses behavioral, emotional, and cognitive components that reflect active age-appropriate student participation in school and their investment in learning-related behavior (Fredricks et al. 2004). As such, understanding early factors that might shape engagement has become a priority in developmental and educational research (Fitzpatrick and Pagani 2013). Among these, early literacy experiences in the home likely plays a foundational role (Fox et al. 2010). Parents are often encouraged by pediatricians and allied professionals to read with their very young children to create meaningful bonding moments, generate curiosity, and foster academic interest (Klass et al. 2024). Parent–child book reading in the first 2 years of life is highly recommended and considered one of the most effective endeavors for promoting language and literacy development (Brown et al. 2022). Such activities, combined with a growth mindset message from parents, lead to positive cognitive outcomes (Tian et al. 2023).

Research has established a net correlation between early childhood precursors to reading and academic achievement through to emerging adulthood (Duncan et al. 2007; Pagani et al. 2010). While achievement is undoubtedly a core component of educational outcomes, cognitive ability explains much of the variation in student performance. However, education extends beyond academics and is increasingly recognized as a psycho-social endeavor. Focusing solely on cognitive skills as outcomes likely overlooks essential self-determining factors that contribute to overall personal and social functioning (Pagani et al. 2010). There remains a dearth of compelling evidence which suggests that early exposure to literacy correlates with later learning-related behaviors (Klass et al. 2024).

Toddler literacy enrichment refers to the variety of activities that expose young children to literacy experiences at home before formal schooling begins (Herbers et al. 2012; Sun et al. 2024). It encompasses interaction with printed materials and early writing activities that support emergent literacy skills (Brown et al. 2022). Literacy-related activities at home, such as looking at books, playing with pencils or pretend writing, or being read to by an adult in the household, represent actionable targets for measuring child exposure.

Children’s proclivity toward language and literacy competence begins in the womb (Partanen et al. 2013). As primary caregivers, parents are highly influential in reinforcing such predispositions, through parent–child interactions related to reading (Conica et al. 2023; Merga and Ledger 2018). Early childhood contact with the printed word represents a continuum of out-of-school reading experiences that facilitate children’s language, reading, and spelling achievement through adulthood (Mol and Bus 2011). Early caregiving experiences can shape the brain’s architecture and consequential behavioral development across the life course (Margolis and Gabard-Durnam 2025). Indeed, the home literacy environment is where children first acquire the language and literacy skills that equip them to interpret, describe, and participate in the world (Liebeskind et al. 2014). During shared reading, parents engage children in conversations that support the development of problem-solving abilities and broader cognitive growth (Shahaeian et al. 2018).

A recent report by the American Academy of Pediatrics indicates that shared reading is essential for early childhood development, school readiness, and long-term educational success (Klass et al. 2024). It fosters foundational skills before formal schooling begins and reflects the home environment and parent–child relationships (Herbers et al. 2012; Mol and Bus 2011). Early literacy helps children access and internalize information from books, conversations, and other educational content. For example, early exposure to reading has been linked to more frequent reading in the future, greater enjoyment and interest in reading, higher vocabulary scores, and improved language and social communication skills (Brown et al. 2022; Merga and Ledger 2018). Consequently, curiosity and vocabulary, reading comprehension, and decoding skills lay the foundation for acquiring and retaining knowledge (Sun et al. 2024). Although this bidirectional process generates a rich knowledge base, which is the hallmark of crystallized intelligence (Hülür et al. 2018), it is plausible that such early family experiences foster the learning-related behavioral mechanisms that shape knowledge acquisition.

Several long-term studies suggest that early home literacy experiences predict later academic outcomes, above and beyond the influence of maternal education. Using data from the Longitudinal Study of Australian Children (n = 4768), Shahaeian et al. (2018) examined the longitudinal association between the frequency of early shared reading and later school achievement in children, independent of family and demographic factors. They found that shared reading between ages 2 and 3 years predicted children’s school achievement directly and indirectly through receptive language and early academic skills especially among low- and middle-income families compared with the higher-income families. It was concluded that books offer unique opportunities to teach children new words and concepts in a systematic way, and this is something that most parents would not be able to do otherwise. Similarly, Lehrl et al. (2019) investigated the longitudinal and differential associations between preschool reading experiences at age 3 years and subsequent achievement outcomes at age 13 years in a study of 554 German youth. They found that book exposure and the quality of verbal interactions regarding mathematics both predicted math achievement in secondary school. Their effects were mediated by early language and arithmetic skills from home literacy exposure. More recently, in a study of several thousand Irish children, Conica et al. (2023) found that informal home literacy and numeracy environments at age 3 years (through playing board and card games, having access to books, and shared reading) predicted domain-specific and cross-domain positive associations with language and numeracy outcomes in youth at ages 5 and 9 years. Thus, a home culture of informal learning that does not actively focus on teaching seems to have positive outcomes for subsequent reading and math achievement.

The long-term behavioral mechanisms behind such benefits remain to be established, especially at school. Early literacy enrichment may demonstrate value by promoting the participatory nature of parent-guided activities, which later supports learning-related behavioral engagement when faced with academic challenges. School engagement comprises behavioral, emotional, and cognitive elements that contribute to active student investment in learning environments (Fredricks et al. 2004). Relatedly, research on cognitive engagement suggests that sustained involvement in such activities is associated with the development of learning-related skills over time (Hülür et al. 2018). This is because active participation often leads to greater exposure to educational content, opportunities for critical thinking, and the application of learned knowledge in various contexts. This likely explains how engagement leads to personal and professional success (Herbers et al. 2012).

Sensitive period theory offers a compelling explanation for the relationship between an enriched literacy environment early in life and later child outcomes. Gee and Cohodes (2021) refer to the sensitive period as a developmental window of heightened neuroplasticity during which specific environmental stimulation has an especially strong effect on later flourishment. Indeed, the first 1000 days of life represent a particularly sensitive window for shaping long-term cognitive, emotional, and physical outcomes (Bhutta et al. 2013). Proper care and enrichment during this period, including early exposure to literacy, shapes brain plasticity and lays the foundation for healthy child development (Berretta et al. 2021). Parental engagement in early stimulation, such as book reading, storytelling, and early writing, provides essential inputs that foster cognitive flexibility, language development, and school readiness. Neuroscience highlights the role of early experiences in shaping the brain, as synaptic connections formed and reinforced through early stimulation are critical for the development of a functional “reading network” in the brain (Gee and Cohodes 2021). This suggests that early interventions can have long-term academic and developmental benefits (Bosseler et al. 2024).

Bronfenbrenner’s bioecological systems model explains how the individual child and microsystemic influences interact and propel development (Jaeger 2017). More specifically, literacy enrichment occurs at the site of transaction between child cognitive processes and value-driven parental social practices. This facilitates developing a predisposition toward social cooperativeness and task-oriented behavior in early childhood that inspires learning-related engagement from childhood through to emerging adulthood (Fitzpatrick and Pagani 2013; Fitzpatrick et al. 2020). In a study spanning three decades, Symonds et al. (2023) found that engagement at school predicted long-term benefits for educational and employment outcomes in adulthood.

This field of study has its methodological flaws. In longitudinal studies on the matter, confound controls have been limited to maternal education and indicators of family income, failing to account for the influence of important individual child and family characteristics. Pre-existing and concurrent confounding variables beyond the maternal education and socio-economic status, such as gestational exposures to neurotoxins, infant temperament, or cognitive factors like toddler working memory skills, can shape both early literacy opportunities and later outcomes. A child with a difficult temperament or poor working memory may struggle with participating in a learning environment, independently of previous experiences in early development. Moreover, children with parents who have a history of mental health challenges may come from households with fewer literacy enrichment opportunities. There are thus pre-existing maternal and child factors of influence beyond maternal education to consider.

Numerous individual and family factors have been shown to influence early literacy experiences and later student engagement. For instance, cognitive ability and maternal education and depression and antisocial behavior have been consistently linked to child language development and academic trajectory outcomes (Lehrl et al. 2019; Shahaeian et al. 2018). Family income, configuration, and dysfunction have also been associated with child behavior and engagement in school (Pagani et al. 2010; Fitzpatrick and Pagani 2013). Therefore, by including these covariates in our models, we reduce potential confounding and competing explanations and optimally isolate the association between early literacy enrichment and later outcomes.

Most studies in this area have controlled for sex as a covariate, comparing boys and girls with each other rather than with same-sex experiences (e.g., Mol and Bus 2011). Growing up as a son or daughter has associated biological influences and gendered social expectations which influence responses to risk and protective factors during human development (Rajhans et al. 2019). Real life does not occur in a vacuum of biological and social experiences, thus justifying stratification by sex allows us to take into account such distinctions (Bornstein et al. 2016). Prior research has demonstrated that boys and girls differ in how they respond to environmental influences (Montgomery et al. 2017). For instance, studies have shown that cognitive ability, parenting, and environmental stimulation can affect children’s behavioral and academic outcomes differently based on biological influences and parental expectations (Tannenbaum et al. 2019). Therefore, sex stratification captures potentially distinct developmental pathways by comparing girls with girls and boys with boys on key variables of interest, rather than assuming a uniform pattern across a human sample (Johnson et al. 2009).

As a first, the purpose of this study is to investigate the long-term associations between early enrichment activities related to parent–child reading in toddlerhood and later student engagement through to the end of high school in a millennial birth cohort. More specifically, we use a prospective longitudinal design to examine whether literacy enrichment at age 2 years has associated benefits for long-term student engagement at ages 6, 12, and 17 years. These age outcomes correspond to key academic transitions at the end of kindergarten, sixth grade, and high school, respectively. We expect that experiences related to early childhood literacy in the family environment will predict favorable long-term associations according to teachers and youth self-reports.

## 2. Methods

### 2.1. Participants

Participants from this IRB-approved investigation are from the Quebec Longitudinal Study of Child Development (QLSCD) birth cohort, coordinated by the *Institut de la Statistique du Québec*. The original sampling, launched to track the growth of typically developing children, consisted of 2837 randomly selected infants born between 1997 and 1998 in all administrative regions of Quebec, Canada. At the inception of the study, 93 selected children were deemed ineligible, 172 were untraceable, 14 were unreachable, and 438 were unreachable or refused participation. Ultimately, 2120 infants were deemed eligible for the initial follow-up from age 5 months onward (http://www.iamillbe.stat.gouv.qc.ca/default_an.htm, accessed on 4 June 2025). Annual follow-ups took place during childhood, and biennial follow-ups occurred during formal schooling. For each data collection wave, informed consent was obtained from the parents. During the school-aged phase, teachers and children also gave consent. For this study, a subsample of 1006 boys and 991 girls with complete data on literacy enrichment at age 2 years was retained. 

### 2.2. Measures: Predictor—Literacy Enrichment (Age 2 Years) 

During early childhood, parents reported on the types of literacy enrichment their child was exposed to in the home (Fitzpatrick and Pagani 2013; Pagani et al. 2010). They were asked how often their child: (a) looked at books, magazines, comics, etc., (b) played with pencils or markers doing real or pretend writing at home, and (c) was read to by an adult of the household. Responses varied on an 8-point Likert scale from “Never or rarely” to “Many times each day”, recoded into “Once a week or less” (=0) or “A few times a week or more” (=1) for each question. A sum of three types of literacy enrichment at home was calculated to create an index ranging from 0 to 3.

### 2.3. Measures: Outcomes—Indicators of Student Engagement (Ages 6, 12, and 17 Years)

Classroom Engagement was reported by homeroom teachers through 7 items at age 6 years (Fitzpatrick et al. 2020; Pagani et al. 2010): follows directions; follows rules and instructions; listens attentively; works neatly and carefully; works independently; works and plays co-operatively with other children; and completes work on time (α_Boys_ = 0.85 and α_Girls_ = 0.84). At age 12 years, homeroom teachers reported on 11 items: follows directions; follows rules; follows instructions; listens attentively; works neatly and carefully; works independently; works co-operatively with other children; completes work on time; participates in class; asks questions when they do not understand; and puts a lot of effort into work (α_Boys_ = 0.92 and α_Girls_ = 0.90). Items were rated on a 5-point Likert scale from “Never” to “Always”. A sum of scores was converted to a scale from 0 to 10, where a higher score represents greater engagement. 

School Engagement was self-reported through four questions at ages 12 and 17 years (Fitzpatrick et al. 2020): (a) “Do you like school?”, (b) “In terms of your school marks, how would you rate yourself compared with other students your age at your school”, (c) “How important is it for you to get good marks?”, and (d) “What is the highest level of schooling that you would like to achieve?” (α_Boys_ = 0.51 and α_Girls_ = 0.53 at age 12 years and α_Boys_ = 0.62 and α_Girls_ = 0.64 at age 17 years). Scores were summed to create a scale from 4 to 18, where a higher score represents greater engagement. 

### 2.4. Measures: Confound Control Variables (Ages 5 Months to 2 Years) 

To rule out pre-existing and concurrent confounding variables between literacy enrichment and later academic engagement, relevant early childhood individual and family-related characteristics were identified based on statistical analyses and the existing literature.

With regard to child-level variables, at age 5 months, mothers reported gestational smoking and substance use (0 = no substance use, 1 = any substance use), whereas premature birth (0 = not preterm, 1 = preterm, <37 weeks) and weight for gestational age (g/weeks) were obtained from medical records. Child temperament problems were reported by mothers at age 1.5 years using the Infant Characteristics Questionnaire (10 items: 0 = below the median, 1 = above the median, α_Boys_ = 0.78 and α_Girls_ = 0.75; Bates et al. 1979) and neurocognitive skills were assessed by trained examiners at age 2 years using the Imitation Sorting Task (Alp 1994).

In terms of potential family-related confounds, maternal antisocial antecedents using the National Institute of Mental Health—Diagnostic Interview Schedule at age 5 months (9 items: 0 = below the median, 1 = above the median; α = 0.46; Robins et al. 1981), maternal education at age 5 months (0 = finished high school, 1 = did not finish high school), and maternal depressive symptoms using the Center for Epidemiologic Studies Depression Scale at age 1.5 years (13 items; 0 = below the median, 1 = above the median, α = 0.82; Radloff 1977) were reported by mothers. Parents reported family dysfunction using the McMaster Family Assessment Device at age 5 months (12 items; 0 = below the median, 1 = above the median, α = 0.88; Epstein et al. 1983), family income at age 2 years (0 = sufficient income, 1 = insufficient income; as defined by the Canadian low-income cut-off of that year provided by Statistics Canada (2022)), and family configuration at age 2 years (0 = intact; 1 = non-intact). 

### 2.5. Data Analytic Procedures 

Descriptive statistics were generated using SPSS (v.29). Long-term prospective linear associations were estimated using ordinary least squares multiple regression in Mplus (v. 8.0), stratified by sex. Indicators of student engagement from ages 6 to 17 years were regressed on literacy enrichment in the home at age 2 years. Adjustments for possible omitted variable bias and competing explanations were accounted for by including pre-existing and concurrent child and family characteristics that could influence predictor or outcome variables. For each outcome, predictor and confound controls were entered simultaneously. 

As this study required data from various sources and waves, some participants have incomplete data, as expected in longitudinal studies. To minimize and correct for attrition bias from incomplete data, we used the Full Information Maximum Likelihood procedure in Mplus with the Maximum Likelihood with Robust standard errors estimator. 

## 3. Results

Table 1 reports descriptive statistics for the predictor, outcome, and confound control variables between the ages of 5 months and 17 years. It is noteworthy that, at age 2 years, more than half of boys and girls were exposed to all three types of literacy enrichment in the home. Fewer than one in ten children were exposed to one or fewer stimulators. The children in this study showed positively skewed classroom and school engagement scores, having relatively high averages from ages 6 to 17 years.

Table 2 documents adjusted unstandardized relationships between literacy enrichment at age 2 years and pre-existing and concurrent child and family confounding variables between the ages of 5 months and 2 years, for boys and girls. For boys, low maternal education (b = −0.09, *p* ≤ 0.05, 95% confidence interval [CI]: −0.30, −0.06) and family dysfunction (b = −0.06, *p* ≤ 0.05, 95% CI: −0.17, −0.01) significantly predicted less literacy enrichment two years later. One family characteristic, maternal depressive symptoms, significantly predicted less literacy enrichment for girls (b = −0.09, *p* ≤ 0.05, 95% CI: −0.17, −0.04). Being born prematurely (b = −0.08, *p* ≤ 0.05, 95% CI: −0.42, −0.04) and scoring less on neurocognitive skills (b = 0.12, *p* ≤ 0.001, 95% CI: 0.05, 0.13) were also associated with lower literacy enrichment for girls. 

Table 3 reports adjusted unstandardized regression coefficients reflecting the relationship between literacy enrichment at age 2 years and indicators of academic engagement from ages 6 to 17 years. Compared to boys with less literacy enrichment in early childhood, boys who were more exposed to literacy stimulation had higher classroom engagement at age 6 years (b = 0.17, *p* ≤ 0.001, 95% CI: 0.25, 0.72) as well as school engagement at ages 12 and 17 years (b = 0.08, *p* ≤ 0.05, 95% CI: 0.07, 0.52 and b = 0.09, *p* ≤ 0.05, 95% CI: 0.05, 0.58, respectively). Girls with more literacy enrichment in early childhood also had higher classroom engagement at age 6 years (b = 0.10, *p* ≤ 0.05, 95% CI: 0.08, 0.50) and school engagement at age 17 years (b = 0.09, *p* ≤ 0.05, 95% CI: 0.10, 0.60) compared with girls who had fewer literacy enrichment.

Several individual and family confounders in early childhood were associated with indicators of academic achievement throughout elementary and high school. Notable is maternal education for boys, where having a mother that had not finished high school significantly reduced classroom engagement at age 6 years (b = −0.13, *p* ≤ 0.05, 95% CI: −1.29, −0.19) and school engagement at age 12 years (b = −0.13, *p* ≤ 0.01, 95% CI: −1.48, −0.40). Unexpectedly, boys with a difficult and unpredictable temperament and those belonging to a non-intact family had higher classroom engagement (b = 0.19, *p* ≤ 0.001, 95% CI: 0.40, 0.94) and school engagement (b = 0.09, *p* ≤ 0.05, 95% CI: 0.11, 1.16) at age 12 years, respectively. For girls, having a mother without a high school diploma also predicted lower classroom (b = −0.18, *p* ≤ 0.001, 95% CI: −1.03, −0.35) and school engagement (b = −0.10, *p* ≤ 0.05, 95% CI: −1.15, −0.22) at age 12 years. Maternal depressive symptoms were consistently associated with lower classroom engagement (b_6 years_ = −0.11, *p* ≤ 0.05, 95% CI: −0.64, −0.10 and b_12 years_ = −0.14, *p* ≤ 0.01, 95% CI: −0.61, −0.18) and school engagement (b_12 years_ = −0.13, *p* ≤ 0.001, 95% CI: −0.96, −0.32), whereas higher neurocognitive skills were associated with higher classroom engagement (b_6 years_ = 0.13, *p* ≤ 0.01, 95% CI: 0.12, 0.40 and b_12 years_ = 0.14, *p* ≤ 0.001, 95% CI: 0.12, 0.36) from ages 6 to 12 years.

## 4. Discussion

Parents are encouraged to read to their very young children with hopes of stimulating an early interest in learning, reading, and written communication (Klass et al. 2024). While there has been research on early stimulation and later academic achievement, few studies have examined whether early parental behaviors in toddlerhood are prospectively associated with learning-related behaviors, such as student engagement, across development. This investigation provides compelling and timely evidence of such associations through to the end of high school in typically developing boys and girls.

Our findings indicate that just over half of children experienced leisure time reading and writing activities at home with adults. This includes looking at books, magazines, or comics, playing with pencils or markers for real or pretend writing, and being read to by an adult in the household. Notably, a similar proportion showed positive student engagement scores.

More specifically, compared with their less stimulated same-sex counterparts, boys who were read to and experienced more exposure to written and printed words were observed to have more classroom engagement by age 6. For every unit increase on the literacy exposure scale, boys experience a 17% standard deviation unit increase in classroom engagement. This means they were proportionately more likely to follow directions, respect rules and instructions, listen attentively, and work cooperatively with other children. Such boys also reported 8% associated increases in overall school engagement, reflected in higher school enjoyment, academic self-perception, motivation to succeed, and educational aspirations, compared with boys who had less early literacy stimulation exposure.

Girls who experienced shared reading and exposure to materials related to reading and writing in toddlerhood had 10% unit increases in classroom engagement, as reported by teachers at ages 6 and 12, compared with girls having had less early exposure. Such girls were more likely to follow directions, listen attentively, and cooperate with kindergarten and sixth grade classmates. Girls also self-reported 9% associated increases in overall school engagement at the end of high school, including more positive perceptions of school, higher academic self-concept, and stronger beliefs in the value of school achievement.

These promising findings highlight the behavioral and motivational benefits of exposure to literacy exposure in toddlerhood and how it shapes developmental trajectories toward success. Specifically, they support prior research suggesting that the early years represent a sensitive period during which enriched environments, such as those involving quality family time, can influence the neural pathways that underpin cognitive, social, and emotional development (Brown et al. 2022; Klass et al. 2024). Consistent exposure to books, storytelling, and writing materials during toddlerhood generates a growth perspective that lays the foundational skills for subsequent classroom and school engagement. 

The outcomes in this study align with conceptualizations of behavioral engagement as learning-related behaviors such as effort, persistence, and participation, thus offering an objective dimension of internal motivation through measurable participation. This aligns with the principles of self-determination theory (Ryan et al. 2019), which inspired conceptualizations of student engagement (Fredricks et al. 2004). Specifically, behavioral engagement reflects how students experience and cultivate autonomy, competence, and relatedness through values in action. By emphasizing behavioral engagement, our study captures a key developmental aspect of learning-related behavior, especially in large-scale, longitudinal research.

Furthermore, developmental pathways are shaped not only by biology, but by a range of microsystemic social influences within the family environment (Jaeger 2017). Parents are primary agents in fostering cognitive activities as influences of socialization and school-related skills and attitudes (Wilder 2013; Woreta 2024). By engaging in early literacy activities, parents lay the groundwork for sustained curiosity and motivation, both key factors that ultimately contribute to the evolution of student engagement across human development.

This study is not without limitations. First, although this study is observational, and our literacy enrichment measure relies on parent reports, which may be subject to social desirability effects. Although imprecise, such reports provide valuable insights into typical early childhood experiences and their associated, long-term benefits. Second, this study is longitudinal, and the findings remain correlational. Consequently, we cannot provide definitive causal connections. Finally, our ability to relate our engagement variable to a standardized achievement outcome was not possible, as this investigation represents a secondary analysis of existing longitudinal data. Nonetheless, self-reported school engagement has been shown to strongly correlate with more objective assessments of academic achievement and is widely recognized as a key driver of long-term success (Symonds et al. 2023).

This study has several chief strengths that provide a well-rounded picture of academic and classroom involvement, attachment, and interest in school, as described by Symonds et al. (2023). First, its longitudinal approach allows for tracking early literacy engagement and its association with student engagement over the childhood life course into emerging adulthood. Second, this study, using millennial birth cohort data, occurred at a time when there was less confounding with child screen time. Obtaining estimates with today’s ubiquitous exposure to technology would have been very challenging. Third, its prospective nature ensured that pre-existing influences were measured before the literacy stimulation predictor, which helped isolate the unique and net contribution of early literacy experiences to subsequent transitions that are key in the academic journey. Finally, the distinction between boys and girls as heterogeneous populations acknowledges their distinct interests, motivations, and contextual experiences, offering deeper insights into how early literacy enrichment shapes engagement across the sexes. These features enhance both the internal and external validity of our findings.

Our observations reinforce the importance of investigating actionable features of early parental influence on child development. As an alternative to screen time, activities at home should integrate structured literacy enrichment activities such as shared reading, storytelling, and early writing experiences to cultivate behavioral engagement in developing cognitive skills. Moreover, policymakers should prioritize investments in early literacy programs, parental education, and accessible reading materials for young children, particularly in underprivileged communities. Finally, pediatricians and allied health professionals play a crucial role in promoting a developmental growth perspective by advising parents and caregivers on approaches to literacy exposure during the sensitive period from birth to the end of toddlerhood in developing behavioral propensities that facilitate crystallized intelligence (Tian et al. 2023). By investing early, the family environment can lay a foundation that supports lifelong interest in developing cognitive skills and personal success.

What is known: Cross-sectional research suggests that enrichment in toddlerhood may promote cognitive skills. There have been few longitudinal studies that have investigated the link between early home literacy activities and long-term student engagement outcomes, which translate to school success.

What this study adds: We found associations between toddler literacy enrichment and later student engagement across key academic transitions, from the end of kindergarten through the end of high school. These observations highlight the lasting influence of early literacy stimulation in home environments on subsequent school engagement, a cornerstone of crystallized intelligence.

## Figures and Tables

**Table 1:** Descriptive statistics for the predictor, outcome, and confound control variables.

		Boys			Girls	
	M (SD)	Categorical Variables (%)	Range	M (SD)	Categorical Variables (%)	Range
Predictor						
Literacy enrichment (2 years)						
0 = No enrichment		1.8			0.2	
1 = One type		8.6			4.5	
2 = Two types		34.9			37.9	
3 = Three types		54.7			57.3	
Outcomes						
Classroom engagement (6 years)	7.94 (2.05)		0.00–10.00	8.77 (1.66)		0.00–10.00
Classroom engagement (12 years)	7.09 (1.77)		0.00–10.00	8.23 (1.41)		0.00–10.00
School engagement (12 years)	13.65 (2.60)		4.00–18.00	14.61 (2.41)		4.00–18.00
School engagement (17 years)	13.15 (2.47)		4.00–18.00	14.33 (2.21)		4.00–18.00
Control variables						
Gestational smoking/substance use (5 months) 1 = Any smoking or substance use		31.8			31.1	
Premature birth (5 months) 1 = <37 weeks		5.2			4.2	
Weight for gestational age (5 months)	56.63 (8.62)		15.23–86.15	55.43 (7.89)		18.47–82.83
Maternal antisocial antecedents (5 months) 1 = Above the median		26.8			25.0	
Maternal education (5 months) 1 = Did not finish high school		15.4			15.2	
Family dysfunction (5 months) 1 = Above the median		47.0			46.2	
Maternal depressive symptoms (1.5 years) 1 = Above the median		45.5			43.7	
Child temperament problems (1.5 year) 1 = Above the median		48.5			48.5	
Family income (2 years) 1 = Insufficient income		18.6			18.9	
Family configuration (2 years) 1 = Non-intact		14.9			13.0	
Child neurocognitive skills (2 years)						
0 = Score of 0		20.9			19.8	
1 = Score of 1		52.2			51.5	
2 = Score of 2		23.9			23.2	
3 = Score of 3		2.9			5.5	

*Notes.* M = mean; SD = standard deviation. Analyses corrected for attrition bias. Data were compiled from the final master file of the Quebec Longitudinal Study of Child Development (1998–2015), *©Gouvernement du Québec, Institut de la statistique du Québec*.

**Table 2:** Unstandardized regression coefficients (standard error) reflecting the adjusted relationship between pre-existing and concurrent child and family characteristics between ages 5 months and 2 years and literacy enrichment at age 2 years.

	b(SE)	
	Literacy Enrichment (2 Years)	
	Boys	Girls
Gestational smoking/substance use (5 months)	0.03 (0.05)	−0.07 (0.04)
Premature birth (5 months)	0.16 (0.11)	−0.23 (0.12) *
Weight for gestational age (5 months)	0.00 (0.00)	0.00 (0.00)
Maternal antisocial behavior (5 months)	0.00 (0.05)	0.05 (0.04)
Maternal education (5 months)	−0.18 (0.07) *	−0.08 (0.06)
Family dysfunction (5 months)	−0.09 (0.05) *	−0.07 (0.04)
Maternal depressive symptoms (1.5 years)	0.01 (0.05)	−0.10 (0.04) *
Child temperament problems (1.5 year)	−0.06 (0.05)	−0.04 (0.04)
Family income (2 years)	−0.01 (0.07)	−0.10 (0.06)
Family configuration (2 years)	−0.03 (0.08)	0.09 (0.06)
Child neurocognitive skills (2 years)	0.01 (0.03)	0.09 (0.02) ***
Adjusted R^2^	0.018 *	0.054 ***

*Notes.* * *p* ≤ .05, *** *p* ≤ .001. Analyses corrected for attrition bias. Data were compiled from the final master file of the Quebec Longitudinal Study of Child Development (1998–2015), *©Gouvernement du Québec, Institut de la statistique du Québec*.

**Table 3:** Unstandardized regression coefficients (standard error) reflecting the adjusted relationship between literacy enrichment at age 2 years and academic engagement from ages 6 to 17 years.

		Classroom Engagement	Classroom Engagement	School Engagement	School Engagement
		6 Years	12 Years	12 Years	17 Years
BOYS	Literacy enrichment (2 years)	0.49 (0.14) ***	0.16 (0.11)	0.29 (0.14) *	0.32 (0.16) *
	Gestational smoking/substance use (5 months)	−0.21 (0.21)	−0.13 (0.18)	0.14 (0.22)	0.15 (0.23)
	Premature birth (5 months)	−0.54 (0.49)	0.20 (0.35)	0.68 (0.53)	−0.34 (0.60)
	Weight for gestational age (5 months)	0.00 (0.01)	0.01 (0.01)	0.02 (0.01)	−0.02 (0.01)
	Maternal antisocial behavior (5 months)	−0.19 (0.22)	−0.40 (0.19) *	−0.39 (0.24)	−0.07 (0.26)
	Maternal education (5 months)	−0.74 (0.34) *	−0.51 (0.28)	−0.94 (0.33) **	−0.51 (0.37)
	Family dysfunction (5 months)	0.06 (0.21)	−0.04 (0.17)	0.13 (0.21)	−0.34 (0.23)
	Maternal depressive symptoms (1.5 years)	−0.08 (0.22)	−0.19 (0.18)	−0.03 (0.22)	−0.03 (0.24)
	Child temperament problems (1.5 years)	−0.12 (0.19)	0.67 (0.16) ***	0.29 (0.21)	0.01 (0.22)
	Family income (2 years)	−0.62 (0.32)	−0.32 (0.26)	−0.56 (0.33)	−0.34 (0.36)
	Family configuration (2 years)	0.20 (0.31)	−0.17 (0.27)	0.64 (0.32) *	0.22 (0.39)
	Child neurocognitive skills (2 years)	0.17 (0.12)	0.08 (0.11)	0.16 (0.15)	0.16 (0.16)
	Adjusted R^2^	0.095 **	0.085 ***	0.051 **	0.033
GIRLS	Literacy enrichment (2 years)	0.29 (0.13) *	0.16 (0.10)	0.22 (0.17)	0.35 (0.15) *
	Gestational smoking/substance use (5 months)	−0.23 (0.17)	−0.28 (0.15)	−0.70 (0.22) ***	−0.23 (0.21)
	Premature birth (5 months)	0.06 (0.47)	0.38 (0.36)	0.26 (0.51)	−0.77 (0.52)
	Weight for gestational age (5 months)	0.00 (0.01)	0.00 (0.01)	0.00 (0.01)	0.00 (0.01)
	Maternal antisocial behavior (5 months)	0.05 (0.18)	−0.05 (0.14)	0.19 (0.22)	0.04 (0.21)
	Maternal education (5 months)	−0.16 (0.24)	−0.69 (0.21) ***	−0.69 (0.28) *	−0.47 (0.29)
	Family dysfunction (5 months)	0.05 (0.15)	−0.08 (0.12)	−0.01 (0.18)	0.11 (0.18)
	Maternal depressive symptoms (1.5 years)	−0.37 (0.16) *	−0.40 (0.13) **	−0.64 (0.20) ***	−0.01 (0.19)
	Child temperament problems (1.5 year)	0.08 (0.14)	−0.07 (0.12)	0.04 (0.18)	−0.14 (0.17)
	Family income (2 years)	−0.08 (0.24)	−0.17 (0.20)	−0.32 (0.30)	−0.86 (0.30) **
	Family configuration (2 years)	−0.35 (0.27)	−0.39 (0.21)	0.03 (0.32)	−0.48 (0.32)
	Child neurocognitive skills (2 years)	0.26 (0.09) **	0.24 (0.07) ***	0.21 (0.13)	−0.01 (0.11)
	Adjusted R^2^	0.065 **	0.151 ***	0.079 ***	0.078 ***

*Notes.* * *p* ≤ 05, ** *p* ≤ .01, *** *p* ≤ .001. Analyses corrected for attrition bias. Data were compiled from the final master file of the Quebec Longitudinal Study of Child Development (1998–2015), *©Gouvernement du Québec, Institut de la statistique du Québec*.

## Data Availability

The data presented in this study are available on request from the *Institut de la Statistique du Québec* (ISQ). The data are not publicly available due to permission of the ISQ.
